# Synchronous ileal carcinoid and primary colonic neoplasms: a case report

**DOI:** 10.4076/1757-1626-2-8317

**Published:** 2009-09-09

**Authors:** Muhammad Imran Aslam, Imen Ben Salha, Salli Muller, John Stuart Jameson

**Affiliations:** 1 Department of General Surgery, University Hospitals of Leicester, Leicester General HospitalGwendolen Road, Leicester LE5 4PWUK; 2 Department of Pathology, University of LeicesterRobert Kilpatrick Building, Leicester Royal Infirmary, PO Box 65, Leicester LE2 7LXUK

## Abstract

Primary colonic tumours with synchronous ileal carcinoid tumours are rare in occurrence and are mainly found incidentally on autopsies or pathological examination of resected surgical specimens. This article describes a case of adenomatous colonic polyps, adenocarcinoma of sigmoid colon and concurrent malignant carcinoid tumour of ileocaecal junction, detected on colonoscopic examination. The radiological staging investigations revealed no distant spread of disease. The patient was effectively treated with subtotal colectomy, resection of terminal ileum, excision of locoregional lymph nodes and the bowel continuity was restored with stapled ileo-rectal anastomosis. This article is as an example of concomitant presence of two types of malignant tumours, effectively managed surgically.

## Case presentation

**Figure 1.**
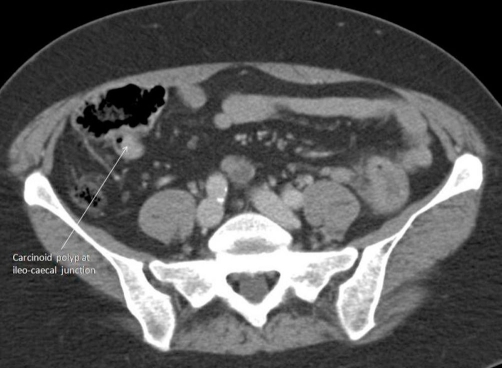
CT scan image showing polypoidal lesion at ileocaecal junction.

**Figure 2.**
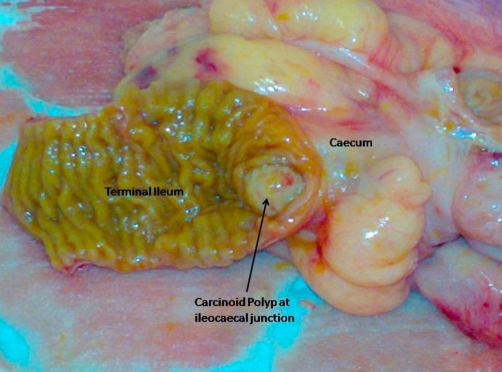
Yellowish white carcinoid lesion at ileocaecal junction. Terminal ileum is opened for demonstration of lesion.

**Figure 3.**
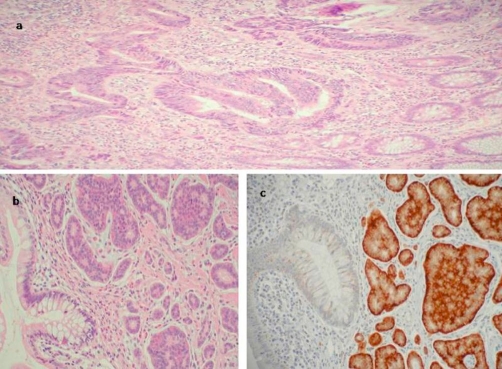
**(a)** Well-differentiated adenocarcinoma of colon infiltrating the serosa (haematoxylin & eosin). **(b)** Multiple solid and acinar groups of cells with finely stippled chromatin (haematoxylin & eosin).The tumour cell are infiltrating beyond the muscularis mucosa. **(c)** Immunohistochemistry of carcinoid: positive synaptophysin staining of tumour cells.

The authors describe the detection and the treatment of synchronous malignant carcinoid tumour of ileocaecal junction and multiple neoplasms of colon. A 67-year-old, Caucasian, white, British female underwent colonoscopic examination as part of the National Bowel Cancer Screening Programme (NBCSP). The colonoscopy revealed multiple polyps of varying morphology in the caecum, transverse, descending and distal sigmoid colon. Terminal ileal intubation showed a non-obstructing polypoidal lesion at ileocaecal junction. The ileal and sigmoid polyps were biopsied and rest excised. The histological features of the ileal lesion were suggestive of malignant carcinoid tumour. The biopsies from the sigmoid polyp showed moderately well differentiated adenocarcinoma and the remainder of the five polyps were low grade tubular/tubulovillous adenomas. Computerised tomographic (CT) scan confirmed the presence of polyp at ileocaecal junction (Figure 1) and the absence of distant spread of carcinomas. The primary adenocarcinoma of the sigmoid colon was a rT3, rN1, rM0 lesion. The patient underwent laparoscopic subtotal colectomy and further excision of terminal 20 cm of ileum for malignant carcinoid tumour (Figure 2). The mesenteric root was carefully inspected and dissected to remove lymph nodes in the mesenteric root close to the superior mesenteric vein. The bowel continuity was restored with the stapled ileorectal anastomosis. The patient made an uneventful recovery from surgery and was discharged on the 5 post operative days. The final histology confirmed the initial diagnosis of an ileal carcinoid tumour (Figure 3c) and a sigmoid adenocarcinoma (Figure 3a,3b). The colonic tumour was staged as pT3 pN2 M0R0. The carcinoid tumour was staged as well differentiated neuroendocrine carcinoma (WHO classification) or pT3 pN2 M0R0 (American Joint Committee on Cancer). Adjuvant chemotherapy for the adenocarcinoma was advised following discussion at the Multidisciplinary Team Meeting.

## Discussion

Primary intestinal carcinoid neoplasms are indolent and often multi-centric. The incidence of synchronous ileal carcinoids in the presence of primary colonic tumours is reported as 1-8% in the literature [1,2]. The synchronous and metachronous midgut carcinoid tumours are usually detected on staging evaluation, surgical exploration, histological examination and autopsies performed for the primary colonic tumours [3,4] The optimum imaging modality depends on whether it is used for detection of primary tumour or for the assessment of the metastasis. Quite contrary to our case, the most midgut carcinoid tumours are not visible on a CT scan, nevertheless the staging CT scan may reveal the presence of lymph nodes enlarged with metastasis from carcinoid cancers [5]. Rarely, the intubation of the terminal ileum on colonoscopy may reveal the incidental ileal carcinoids as described in this case report. For tumours >1 cm in size, a standard surgical resection of primary carcinoid tumour with locoregional lymphadenectomy is appropriate [6]. This was achieved by subtotal colectomy, excision of terminal ileum and the dissection of lymph nodes in the mesenteric root.

## Conclusion

The concomitant presence of primary colonic adenocarcinoma and the ileal carcinoid cancer is a rare phenomenon. In addition to the resection of colonic tumours, the surgery for concomitant carcinoid cancers should involve resection of primary carcinoid tumour with locoregional lymphadenectomy.

## Abbreviations

CT: computerised tomography
NBCSP: National Bowel Cancer Screening Programme
WHO: World Health Organisation

## References

[bib-001] Tse V, Lochhead A, Adams W, Tindal D (1997). Concurrent colonic adenocarcinoma and two ileal carcinoids in a 72-year-old male. Aust N Z J Surg.

[bib-002] Chemli S, Dhouib RS, Mrad K, Ben Mansour Z, Cheour H, Ben Romdhane K, Bouchoucha S (2007). Synchronous association of ileal carcinoid and colorectal carcinoma. A case report. Tunis Med.

[bib-003] Rivadeneira DE, Tuckson WB, Naab T (1996). Increased incidence of second primary malignancy in patients with carcinoid tumors: case report and literature review. J Natl Med Assoc.

[bib-004] Brown NK, Smith MR (1973). Neoplastic diathesis of patients with carcinoids. Cancer.

[bib-005] Lotlikar U, Fogler R, Novetsky AD, Yoon NY (1982). Concurrent colonic carcinoma and small-bowel carcinoid tumor. Case reports and review of the literature. Dis Colon Rectum.

[bib-006] Falconi M, Bettini R, Scarpa A, Capelli P, Pederzoli P (2001). Surgical strategy in the treatment of gastrointestinal neuroendocrine tumours. Ann Oncol.

